# Prevalence of High-Risk Human Papillomavirus and Associated Risk Factors Among Individuals With Head and Neck Cancer in a National Referral Center in Tanzania

**DOI:** 10.1200/GO-25-00596

**Published:** 2026-03-17

**Authors:** Aslam Nkya, Mary Jue Xu, Godfrey Sama Philipo, Summaiya Haddadi, Dianna Ng, Beatrice Paul Mushi, Atuganile Edward Malango, Erick Philip Magorosa, Alan Paciorek, Li Zhang, Sikudhani Muya, Patrick Ha, Edwin Liyombo, Enica Richard, Willybroad Massawe, Joel M. Palefsky, Sue S. Yom, Elia John Mmbaga, Katherine Van Loon

**Affiliations:** ^1^ Department of Otorhinolaryngology, Muhimbili National Hospital, Dar es Salaam, Tanzania; ^2^ Department of Otolaryngology-Head and Neck Surgery, University of California San Francisco, San Francisco, CA; ^3^ The College of Surgeons of East, Central and Southern Africa (COSECSA), Arusha, Tanzania; ^4^ Department of Epidemiology and Biostatistics, Muhimbili University of Health and Allied Sciences, Dar es Salaam, Tanzania; ^5^ Department of Pathology, Memorial Sloan Kettering Cancer Center, New York, NY; ^6^ Department of Pathology, Muhimbili National Hospital, Dar es Salaam, Tanzania; ^7^ Department Epidemiology and Biostatistics and Department of Medicine, University of California San Francisco, San Francisco, CA; ^8^ Division of Hematology/Oncology, Department of Medicine, University of California San Francisco, San Francisco, CA; ^9^ Department of Radiation Oncology, Ocean Road Cancer Institute, Dar es Salaam, Tanzania; ^10^ Department of Otorhinolaryngology, Muhimbili University of Health and Allied Sciences, Dar es Salaam, Tanzania; ^11^ Division of Infectious Disease, Department of Medicine, University of California San Francisco, San Francisco, CA; ^12^ Department of Radiation Oncology, University of California San Francisco, San Francisco, CA

## Abstract

**PURPOSE:**

While the prevalence of human papillomavirus (HPV)–associated head and neck squamous cell carcinoma (HNSCC), specifically in the oropharynx subsite, is increasing in western populations, limited data exist on prevalence in sub-Saharan Africa. This study describes the prevalence and associated risk factors of HPV association among individuals with HNSCC presenting to Muhimbili National Hospital, the largest public tertiary referral center in Tanzania, a country in East Africa.

**METHODS:**

Participants with HNSCC presenting between July 2020 and June 2022 were prospectively recruited in this cross-sectional study. Tumor samples were tested for HPV DNA polymerase chain reaction (PCR) and p16 immunohistochemistry (IHC). Multivariate logistic regression models assessed potential risk factors for HPV.

**RESULTS:**

Among 121 individuals diagnosed with HNSCC, the median age was 55 years (IQR, 47-68 years), 76.0% (92/121) were male, and 10.9% (13/119 tested) were HIV positive. Among all cases of HNSCC, the prevalence of HPV was 14.6% (95% CI, 7.5 to 21.6) using PCR testing and 23.6% (95% CI, 15.5 to 31.7) using p16 IHC as a surrogate marker. For the oropharynx subsite, 23.1% (95% CI, 0.2 to 46.0) of specimens using PCR testing and 28.6% (95% CI, 4.9 to 52.2) using p16 IHC tested positive (Kappa 3.0 [95% CI, –51.1 to 57.1]; McNemar *P* value = .66). Factors independently associated with HPV positivity were oropharynx subsite (odds ratio [OR], 6.4 [95% CI, 1.0 to 40.6], *P* = .048), nonsmokers (OR, 6.4 [95% CI, 1.1 to 35.4], *P* = .035), and unmarried individuals (OR, 10.6 [95% CI, 2.4 to 47.3], *P* = .002). HPV association in HNSCC did not significantly differ by HIV status or sexual activity.

**CONCLUSION:**

HPV association in HNSCC, specifically of the oropharyngeal subsite, is lower in this cohort from Tanzania compared with western populations. Additional regional data are needed to inform clinical care guidelines and vaccination policies.

## BACKGROUND

Head and neck cancers (HNC) are the 7th most common globally.^1^ While overall rates are declining, the incidence of human papilloma virus (HPV)–associated squamous cell carcinoma of the oropharyngeal subsite increased 225% between 1988 and 2004 in the United States, now exceeding the incidence of HPV-associated cervical cancer.^2-4^ These cancers are attributed to high-risk HPV strains.^5,6^ A survival advantage is imparted by HPV association with a 82% 3-year overall survival for American Joint Committee on Cancer 7th edition stage III/IV tumors compared with 57% for HPV-negative oropharyngeal squamous cell carcinomas (OPSCC).^3^ This difference prompted revising cancer staging guidelines, down-staging HPV-associated OPSCC, and de-escalating treatment to minimize toxicity.^3,7-9^ Early data also suggest that the survival benefit may apply to HPV-associated head and neck squamous cell carcinomas (HNSCC) of other subsites.^10^

**CONTEXT**

**Key Objective**
To understand the prevalence and risk factors for human papillomavirus (HPV) association among individuals with head and neck squamous cell carcinoma (HNSCC) in Tanzania.
**Knowledge Generate**
Among all HNSCC, the prevalence of HPV was 14.6% with polymerase chain reaction (PCR) testing and 23.6% with p16 immunohistochemistry (IHC). For the oropharynx subsite, 23.1% of specimens using PCR testing and 28.6% using p16 IHC tested positive (Kappa 3.0, 95% CI, –51.1 to –57.1; McNemar *P* value = .66). The oropharynx subsite, nonsmokers, and unmarried individuals were factors significantly associated with HPV-positive cancers.
**Relevance**
The testing discordance between p16 and HPV PCR and lower attributable fraction regionally suggested that HPV PCR may be favored as the diagnostic for HPV association in head and neck cancers regionally.

While recognition of HPV-associated HNC specifically has changed clinical paradigms in Western populations,^8,9^ less is known about the rates of HPV-associated HNSCC in sub-Saharan Africa. This region has the greatest burden of HPV-driven cervical cancer and the highest prevalence of HIV infection, a risk factor for HPV-driven malignancies.^11,12^ Two international studies, a systematic review and cross-sectional analysis of pathology specimens from 29 countries, noted global rates of HPV association in HNSCC to be 31.5% and 12.2% and rates of HPV-associated OPSCC to be 45.8% and 22.4%, respectively.^13,14^ These studies, however, included no data from sub-Saharan Africa on oropharyngeal cancers and limited data from other head and neck subsites. A subsequent systematic review focused on sub-Saharan Africa including 31 studies and 3,850 patients noted HPV polymerase chain reaction (PCR) positivity was 15.3% among all HNSCC subsites with the highest proportion among nasopharyngeal cancers (16.5%); additionally, p16 immunohistochemistry (IHC) positivity, a surrogate marker for HPV association most validated in the oropharynx, was 13.6% among all HNSCCs with the highest proportion among oropharyngeal cancers (20.3%).^15^ Most studies retrospectively examined tissue samples collected with heterogeneous methods for analyses and few delineated risk factors.

Tanzania is an East African country of 55 million people with a 5% prevalence of HIV infection.^16-18^ Like other sub-Saharan African countries, HPV diagnostics for HNC are not currently integrated into routine care.^19^ Consequently, rates of HPV-associated HNC and the applicability of data from North American and European populations are unknown. This study aims to describe the prevalence of HPV-associated HNC and associated risk factors to inform how HPV testing should be adapted for care and in regional guidelines.^19^ Understanding the prevalence of HPV-associated HNC and risk factors in sub-Saharan Africa has implications across the cancer care continuum including patient prognosis and treatment, national treatment guidelines, resource allocation, and vaccine policy.

## METHODS

### Study Design and Setting

This cross-sectional study consecutively recruited participants diagnosed with HNSCC presenting to the Department of Otorhinolaryngology at Muhimbili National Hospital (MNH), Tanzania's largest tertiary referral center. Eligible participants were ≥18 years, had clinically suspected HNSCC, and were willing to undergo biopsy, interview, and HIV testing. Recruitment occurred from July 2020 to June 2022 and enrolled after informed consent in English or Swahili, per participant preference.

### Data Collection

A trained, culturally matched research coordinator conducted structured interviews in Swahili. The survey (Data Supplement A) incorporated (1) tobacco and alcohol use surveys adapted for an East African population,^20,21^ (2) the US National Health and Nutritional Examination Surveys extensively used to assess cancer risk factors,^22-24^ and (3) sexual history questions.^24^

Aligned with standard care, participants were asked about history of HIV infection. Participants not been tested within the preceding 12 months were referred to the Clinical Treatment Center for HIV care. Under the Tanzanian HIV testing guidelines, participants underwent voluntary counseling, testing using the Abbott ARCHITECT ELISA-based assay, and treatment if indicated at no cost.^25^

Participants underwent a standard diagnostic biopsy, including either fine needle aspiration (FNA)^26^ or incisional versus excisional biopsy of the primary tumor or metastases. Cell block material was collected in formalin. A hematoxylin and eosin (H&E) section from formalin fixed paraffin embedded (FFPE) blocks was reviewed by a pathologist, confirming presence of malignancy. p16 IHC testing, a surrogate marker for HPV association among HNSCC, was performed following this confirmation.

### p16 IHC Testing and Analysis

p16 IHC was performed in Tanzania using the Roche p16 CINtech following validation on previously confirmed samples. Two pathologists reviewed the IHC stain result using established criteria.^27^ The results were interpreted onsite with telepathology support for confirmation and rectifying ambiguity.

### HPV PCR Testing and Analysis

The PCR assay selected was lower cost and less resource intensive than in-situ hybridization assays, therefore better suited for resource-constrained health systems. HPV DNA PCR was performed using the Atila Biosystems AmpFire HPV Screening 16/18/HR assay, which leverages novel isothermal, multiplex amplification coupled with real-time fluorescent detection (Mountain View, CA) to detect most high-risk genotypes (16,18,31,33,35,39,45,51,52,56,58,59,66,68).^28^ This assay uses formalin-fixed paraffin-embedded tissue and validated on tissue biopsy and FNA samples.^28,29^ A 10-µm FFPE slice either from the surgical biopsy or an FNA cell block was used per protocol. Valid tests required internal positive and negative controls. Samples with nonconfirmatory results were assessed once more. The results with inadequate specimen amount or quality were considered invalid and excluded for analyses pertaining to HPV association. Testing was performed in Tanzania.

### Data Analysis

We considered HPV PCR as the gold standard for HPV testing given that p16 IHC is a surrogate marker for HPV infection, mechanistically downstream of an HPV infection and cellular integration.^27^ Agreement of IHC with PCR results was assessed using percent concordance, sensitivity, specificity, positive predictive value (PPV), and negative predictive value (NPV). Cohen's Kappa statistic quantified the agreement, with interpretation thresholds defined as 1%-20% slight concordance, 21%-40% fair concordance, 41%-60% moderate concordance, 61%-80% substantial concordance, and 81%-100% strong concordance. McNemar's chi-squared test evaluated whether the ratio of discordant proportions (false positives *v* false negatives) equals one. Summary statistics with asymptotic normal CI described the prevalence of HPV in HNSCC. Tests of potential predictors of HPV were based on PCR results. Pearson's chi-squared and Kruskal-Wallis tests assessed association between categorical and continuous variables, respectively, and HPV-associated HNSCC. Multivariable logistic regression modeling assessed the Tanzanian population-specific risk factors and previously published risk factors, including HIV status, HNC subsite, treatments, demographics, alcohol consumption, tobacco consumption, oral health, sexual activity, household exposures, and occupational exposures. The final model started with each potential risk factor from a univariate model that showed suggestion of an association with HPV status (*P* value <.10) and then using backward stepwise selection to retain those variables that are independently statistically significantly associated with HPV status (*P* value <.05). A one-sample and one-sided z-test compared the prevalence rate of HPV-positive patients estimated to a comparable prevalence rate in the United States.^30^ All analyses were performed with Stata (version 18.0). Statistical significance was defined as *P* value <.05.

## RESULTS

### Patient Demographics and Disease Characteristics

Among 121 individuals diagnosed with HNSCC, the median age was 55 years (IQR, 47-68 years) and 76.0% (92/121) were male (Table 1). The most common occupation was agriculture (29.8%, 36/121), 69.0% (80/116) were married, and 28.2% (33/117) completed a secondary education or more. In univariate analyses, participants with HPV-associated HNSCC were more likely female (odds ratio [OR], 3.32 [95% CI, 1.03 to 10.65], *P* = .044) and to have completed at least secondary education (OR, 3.56 [95% CI, 1.10 to 11.47], *P* = .034). Participants with HPV-negative HNSCC were more likely to be married (OR, 5.22 [95% CI, 1.56 to 17.43], *P* = .007).

**TABLE 1:** Patient Characteristics by HPV PCR Status

Demographic Factor	Total, No. (%)	Positive, No. (%)	Negative, No. (%)	Unknown, No (%)	*P* ^a^
	121 (100)	14 (12)	82 (68)	25 (21)	
Age, median (IQR)	55 (47-68)	54 (45-63)	55 (45-68)	57 (53-70)	.442
Sex					.037
Male	92 (76)	7 (50)	63 (77)	22	
Female	29 (24)	7 (50)	19 (23)	3	
Marital status					.004
Single	12 (10)	1 (7)	8 (10)	3	
Married	80 (69)	5 (36)	58 (74)	17	
Divorced	12 (10)	4 (29)	4 (5)	4	
Widow/widower	12 (10)	4 (29)	8 (10)	0	
Unknown	5	0	4	1	
Education					.205
No formal	6 (5)	0 (0)	4 (5)	2	
Some primary	21 (18)	4 (29)	15 (19)	2	
Primary	52 (44)	3 (21)	36 (46)	13	
Some secondary	5 (4)	0 (0)	5 (6)	0	
Secondary	16 (14)	4 (29)	8 (10)	4	
Form six	2 (2)	0 (0)	2 (3)	0	
Tertiary	15 (13)	3 (21)	8 (10)	4	
Unknown	4	0	4	0	
Job type					.301
None	7 (6)	1 (7)	6 (7)	0	
Tech	17 (14)	4 (29)	11 (13)	2	
Cleric	5 (4)	1 (7)	4 (5)	0	
Sales	11 (9)	1 (7)	8 (10)	2	
Skilled	16 (13)	0 (0)	12 (15)	4	
Unskilled	19 (16)	2 (14)	12 (15)	5	
Domestic	10 (8)	3 (21)	5 (6)	2	
Agriculture	36 (30)	2 (14)	24 (29)	10	

Abbreviations: HPV, human papillomavirus; PCR, polymerase chain reaction.

^a^ Pearson chi-squared or Kruskal-Wallis tests are calculated using only known values; frequencies of unknown values are shown for completeness.

The most common HNSCC subsites included larynx, hypopharynx, and nasopharynx (Table 2). Oropharynx comprised 13.2% (16/121) of cases. Seventy-five percent of cases (91/121) were late-stage disease and 88.9% (105/118) were recommended chemoradition. There was no evidence of statistically significant imbalances between HPV-positive and HIV-negative patients with respect to subsite, stage, or recommended treatment.

**TABLE 2:** Disease Characteristics by HPV PCR Status

Disease Characteristic	Total, No. (%)	Positive, No. (%)	Negative, No. (%)	Unknown, No. (%)	*P* ^a^
	121 (100)	14 (12)	82 (68)	25 (21)	
Head and neck subsite					.208
Tongue	1 (<1)	1 (7)	0 (0)	0	
Floor of mouth	1 (<1)	0 (0)	1 (1)	0	
Nasopharynx	17 (14)	2 (14)	14 (17)	1	
Oropharynx, NOS	4 (3)	0 (0)	4 (5)	0	
Soft palate	2 (2)	1 (7)	0 (0)	1	
Tonsil	5 (4)	1 (7)	3 (4)	1	
Base of tongue	5 (4)	1 (7)	3 (4)	1	
Hypopharynx	36 (30)	4 (29)	25 (30)	7	
Larynx, NOS	9 (7)	0 (0)	4 (5)	5	
Supraglottis	10 (8)	0 (0)	8 (10)	2	
Glottis	17 (14)	3 (21)	10 (12)	4	
Subglottis	1 (<1)	0 (0)	1 (1)	0	
Paranasal sinus	10 (8)	1 (7)	6 (7)	3	
Other subsite	3 (2)	0 (0)	3 (4)	0	
Stage overall					.462
I	16 (13)	3 (21)	12 (15)	1	
II	14 (12)	1 (7)	11 (13)	2	
III	24 (20)	4 (29)	12 (15)	8	
IV	67 (55)	6 (43)	47 (57)	14	
Tumor stage—AJCC 7th edition					.317
I	19 (16)	3 (21)	15 (18)	1	
II	26 (21)	3 (21)	18 (22)	5	
III	28 (23)	5 (36)	14 (17)	9	
IV	48 (40)	3 (21)	35 (43)	10	
Nodal stage—AJCC 7th edition					.990
0	42 (35)	5 (36)	27 (33)	10	
I	26 (22)	3 (21)	17 (21)	6	
II	41 (34)	5 (36)	29 (36)	7	
III	11 (9)	1 (7)	8 (10)	2	
Unknown	1	0	1	0	
Metastases stage—AJCC 7th edition					.768
0	102 (86)	12 (86)	66 (83)	24	
I	17 (14)	2 (14)	14 (18)	1	
Unknown	2	0	2	0	
Primary treatment recommended					.414
Surgery	7 (6)	0 (0)	5 (6)	2	
Radiation	5 (4)	0 (0)	4 (5)	1	
Chemotherapy	1 (<1)	0 (0)	0 (0)	1	
Chemoradiation	105 (89)	14 (100)	70 (89)	21	
Unknown	3	0	3	0	
Primary treatment intent					.943
Curative	43 (36)	5 (36)	29 (37)	9	
Palliative	75 (64)	9 (64)	50 (63)	16	
Unknown	3	0	3	0	

Abbreviations: AJCC, American Joint Committee on Cancer; HPV, human papillomavirus; NOS, not otherwise specified; PCR, polymerase chain reaction.

^a^ Pearson chi-squared tests are calculated using only known values.

### Human Papillomavirus PCR and p16 IHC Assessment

Using HPV PCR, the prevalence of HPV association was 14.6% (95% CI, 7.5 to 21.6) among all samples and 23.1% (95% CI, 0.2 to 46.0) in the oropharynx. HPV positivity was in 50.0% (1/2) of oral cavity, 12.5% (2/16) of nasopharynx, 23.1% (3/13) oropharynx, 13.8% (4/29) hypopharynx, 11.5% (3/26) larynx, 14.3% (1/7) paranasal sinus, and no (0/3) other subsite specimens (Table 2). Of 14 HPV PCR-positive specimens, 11 were HPV16 genotype, two specimens were of the HPV18 genotype (one nasopharynx, one larynx), and one was another high-risk strain (hypopharynx). The remaining samples included 82 specimens that tested negative and 25 with invalid results or inadequate tissue quantity.

Using p16 IHC, the prevalence of HPV association was 23.6% (95% CI, 15.5 to 31.7) among all samples and 28.6% (95% CI, 4.9 to 52.2) in the oropharynx. p16 IHC positivity was in 0% (0/2) of oral cavity, 21.4% (3/14) nasopharynx, 28.6% (4/14) oropharynx, 17.6% (6/34) hypopharynx, 16.7% (5/30) larynx, 55.6% (5/9) paranasal sinus, and 66.7% (2/3) other subsite specimens.

The prevalence estimate of 23.1% using PCR testing and 28.6% using p16 IHC in the oropharynx subsite in this cohort were statistically significantly lower than the comparable attributable fraction (AF) of 66% from previous publications in the United States (*P* = .001 and .002, respectively).^30^

### Human Papillomavirus PCR and p16 IHC Concordance

For all HNSCC, 91 samples had definitive results for both tests of HPV, with concordance among 69.2% (n = 63) of the samples (95% CI, 59.8 to 78.7; Appendix Table A1). The sensitivity of p16 IHC compared with the gold standard HPV PCR was 33.3% (6.7%-60.0%), specificity 74.7% (65.1%-84.3%), PPV 16.7% (1.8%-31.6%), and NPV 88.1% (80.3%-95.8%; Appendix Table A2).

For OPSCC samples, eight of 13 samples had concordant results (61.5%, 35.1%-88.0%; Appendix Table A1). The sensitivity of p16 IHC compared with HPV PCR was 33.3% (0%-86.7%), specificity 70.0% (41.6%-98.4%), PPV 25.0% (0%-67.4%), and NPV 77.8% (50.6%-100%; Appendix Table A2).

### Lifestyle Exposures and Risk Factors

Among all individuals with HNSCC, 16.7% (20/120) reported current tobacco use and 30.0% (36/120) prior use (Table 3). Regarding alcohol use, 15.0% (18/120) reported current use and 35.0% (42/120) prior use. There's a suggestion that participants with HPV-positive HNSCC were more likely never-smokers (OR, 4.52 [95% CI, 0.94 to 21.70], *P* = .059). There were no significant differences between individuals with HPV-positive and HPV-negative HNSCC regarding sexual activity, occupational, nor oral health exposures (Table 3, Appendix Tables A3 and A4). Of note, 31.1% (31/100) of all participants reported history of oral sex. No significant difference in HPV association in HNSCC was noted by HIV status (Table 3).

**TABLE 3:** Lifestyle Exposures by HPV PCR Status

Lifestyle Factor	Total, No. (%)	Positive, No. (%)	Negative, No. (%)	Unknown, No. (%)	*P* ^a^
	121 (100)	14 (12)	82 (68)	25 (21)	
Tobacco use					.109
Current	20 (17)	0 (0)	11 (13)	9	
Former	36 (30)	2 (15)	26 (32)	8	
Never	64 (53)	11 (85)	45 (55)	8	
Unknown	1	1	0	0	
Tobacco pack years among users, median (IQR)	18 (7-31)	2 (2-2)	12 (6-26)	22 (12-62)	.039
Household tobacco use	40 (34)	4 (29)	27 (34)	9	.704
Alcohol use					.928
Current	18 (15)	2 (14)	12 (15)	4	
Former	42 (35)	4 (29)	27 (33)	11	
Never	60 (50)	8 (57)	42 (52)	10	
Unknown	1	0	1	0	
Alcohol count years among users, median (IQR)	98 (48-166)	51 (36-63)	124 (60-172)	93 (34-120)	.125
HIV status					.744
Negative	106 (89)	12 (86)	71 (89)	23	
Positive	13 (11)	2 (14)	9 (11)	2	
Unknown	2	0	2	0	
Sexually active	120 (99)	14 (100)	82 (100)	24	NC
Age at first sexual encounter, median (IQR)	19 (18-20)	20 (18-22)	19 (18-20)	18 (16-20)	.188
Sexually transmitted disease	28 (28)	4 (40)	16 (24)	8	.265
Vaginal sex	119 (99)	14 (100)	81 (99)	24	.678
Number vaginal partners 6 months, median (IQR)	1 (1-3)	1 (1-3)	1 (1-2)	2 (1-3)	.492
Number vaginal partners lifetime, median (IQR)	5 (3-8)	4 (3-7)	5 (3-8)	6 (4-11)	.855
Oral sex	31 (31)	3 (30)	22 (31)	6	.927
Number oral partners 6 months, median (IQR)	1 (0-2)	1 (0-2)	1 (0-2)	2 (0-2)	.896
Number oral partners lifetime, median (IQR)	2 (1-4)	3 (1-4)	2 (1-4)	2 (2-4)	.933

Abbreviations: HPV, human papillomavirus; PCR, polymerase chain reaction.

^a^ Pearson chi-squared or Kruskal-Wallis tests are calculated using only known values.

### Association of Risk Factors With HPV Association in HNSCC

In univariate regression analyses, female sex (OR, 3.32 [95% CI, 1.03 to 10.65], *P* = .044) and nonmarried status (OR, 5.22 [95% CI, 1.56 to 17.43], *P* = .007) were associated with HPV positivity in HNSCC (Table 4). Additionally, there is a suggestion that individuals reporting no history of tobacco use had a higher rate of HPV positivity (OR, 4.52 [95% CI, 0.94 to 21.70], *P* = .059). People with more education had a higher rate of HPV positivity (OR, 3.56 [95% CI, 1.10 to 11.47], *P* = .034). Overall, there was no significant evidence of an association between HIV status and HPV status (1.31, 0.25 to 6.84, *P* = .745). However, on additional subanalyses by sex (Appendix Table A5), men who were HIV positive had a higher rate of HPV compared with men who were HIV negative (33% *v* 8%), while women who were HIV positive had a lower rate of HPV compared with women who were HIV negative (0% *v* 33%). These two associations in opposite directions were masked in the overall data null result that grouped men and women together. However, the small sample sizes of these subgroups make it impossible to further test for interaction in the multivariate models.

**TABLE 4:** ORs of HPV Association According to PCR Among All Patients With HNSCC

Exposure	Level	Univariate Analysis		Multivariate Analysis	
		OR (95% CI)	*P*	OR (95% CI)	*P*
HIV status	Positive	1.31 (0.25 to 6.84)	.745		
	Negative	1	Reference		
Sex	Female	3.32 (1.03 to 10.65)	.044		
	Male	1	Reference		
Marital status	Single^a^	5.22 (1.56 to 17.43)	.007	10.58 (2.37 to 47.25)	.002
	Married	1	Reference	1	Reference
Residence	Dar es Salaam	1.88 (0.60 to 5.92)	.279		
	Outside Dar es Salaam	1	Reference		
Cooking fuel	Firewood indoors	0.46 (0.14 to 1.48)	.192		
	Other fuel	1	Reference		
Toothbrush evenings	Yes	2.42 (0.74 to 7.94)	.144		
	No	1	Reference		
Tobacco use	Never	4.52 (0.94 to 21.70)	.059	6.36 (1.14 to 35.41)	.035
	Current or past	1	Reference	1	Reference
Alcohol count-years	+2 drink-years	0.99 (0.97 to 1.01)	.243		
Age started sex	+1 year	1.17 (0.92 to 1.48)	.194		
Dysphagia first	Yes	1.34 (0.41 to 4.42)	.628		
	No	1	Reference		
Primary subsite	Oropharynx	1.96 (0.47 to 8.27)	.358	6.41 (1.01 to 40.60)	.048
	Other subsite	1	Reference	1	Reference
Education completed	Secondary to tertiary	3.56 (1.10 to 11.47)	.034		
	None to some secondary	1	Reference		

Abbreviations: HPV, human papillomavirus; OR, odds ratio; PCR, polymerase chain reaction.

^a^ Marital status single includes single, divorced, or widowed patients.

Given its established association in previous studies, the oropharynx subsite was included in multivariate analyses despite showing no evidence in univariate analyses (Table 4). Other factors for the final multivariate model were selected from univariate models showing at least a suggestion of association: sex (*P* = .044), marital status (*P* = .007), toothbrush use evenings (*P* = .144), current or past tobacco use (*P* = .059), age at first sexual activity (*P* = .194), and education (*P* = .034). Factors with an independent statistically significant association with HPV status were retained in the final model and represented the best data fit. Marital status was retained, but not sex because they were collinear; most men were married (75.9%), and most women were single (51.7%). Tobacco use was retained, but not education because they were collinear; the majority of people who completed at least a secondary education were never-smokers (69.7%) and majority of people with less education were smokers currently or in the past (52.9%).

**FIG A1:**
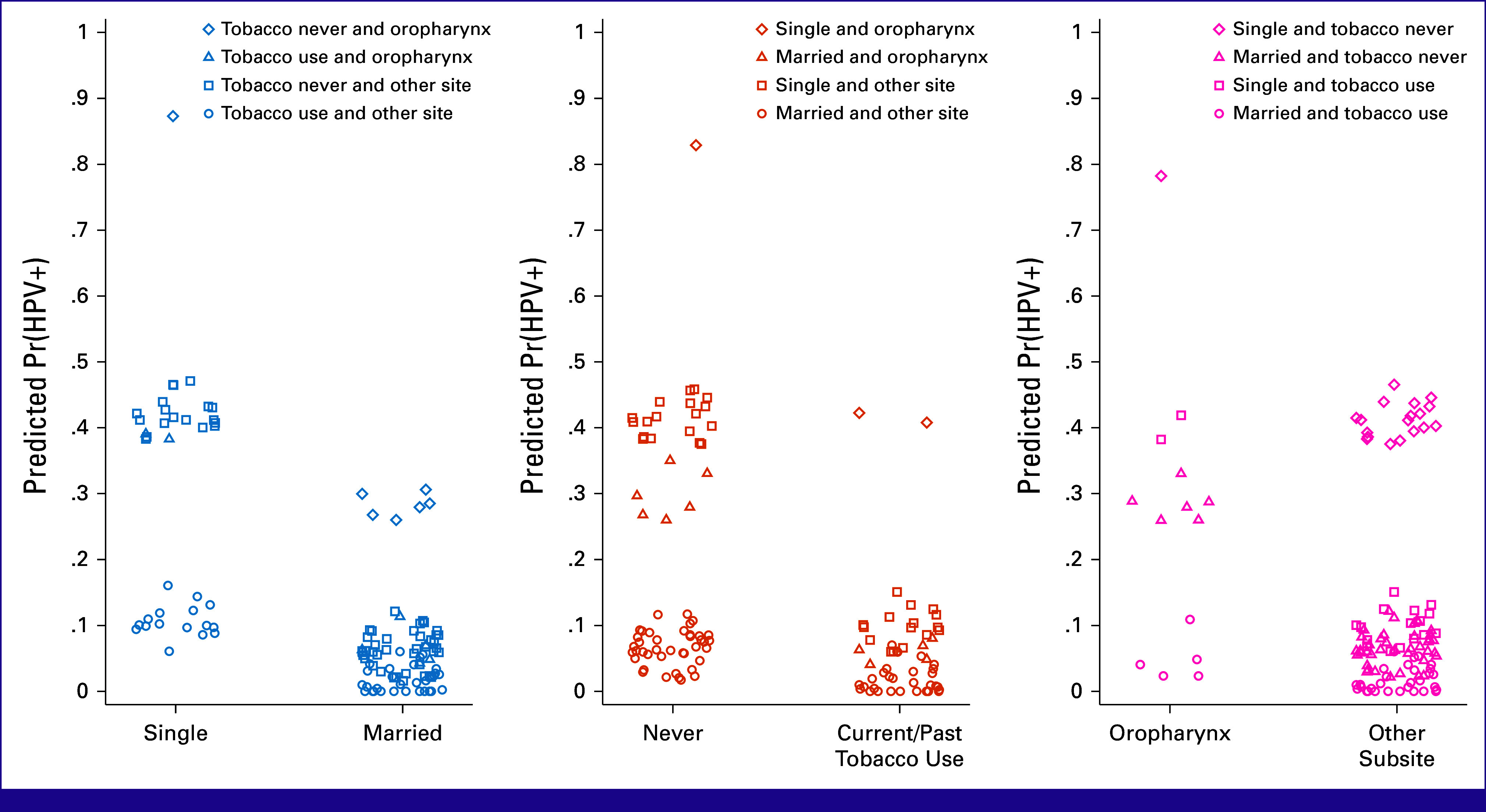
Distribution of predicted probability of HPV-PCR positivity by characteristic. HPV, human papillomavirus.

In the final multivariate regression model, the oropharynx subsite (OR, 6.41 [95% CI, 1.01 to 40.60], *P* = .048), never-smokers (OR, 6.36 [95% CI, 1.14 to 35.41], *P* = .035), and nonmarried individuals (OR, 10.58 [95% CI, 2.37 to 47.25], *P* = .002) had higher adjusted odds of HPV-PCR positivity. Most patients (75%) had a low predicted probability of being HPV positive in the range of 1%-10% because they had at least two protective factors: being married, current or past tobacco use, and primary subsite other than oropharynx (Appendix Fig A1). The other quarter of patients had higher predicted probabilities in the range of 30%-82% and the largest absolute increases in the range of 41%-52% because they had at least two of the risk factors: being single, never used tobacco, and oropharynx subsite.

## DISCUSSION

This cross-sectional study supplements limited literature on HPV-associated HNSCC cancer epidemiology in sub-Saharan Africa.^15^ The proportions in this cohort from Tanzania were significantly lower than those observed in Western populations.^30^ HPV16 was the most common genotype among all HNSCC and for those of the oropharynx. Among samples with results for both p16 IHC and HPV PCR, 31% of the samples were not concordant between tests among all HNSCC and 38% among the oropharyngeal subsite samples, indicating a need to validate HPV diagnostics for HNC within new populations.^31,32^ Finally, in this cohort, we find that HPV-associated HNSCC is associated with the oropharynx subsite, nonsmokers, and nonmarried individuals but with no significant association with HIV status, replicating some risk factors seen in Western populations.^4^

The prevalence patterns seen in this study mirror limited studies from sub-Saharan African countries. Similar to a systematic review of HPV-associated HNSCC in sub-Saharan Africa, the proportion of HPV-associated OPSCC was greater than that of HPV-associated HNSCC overall and below 20%-30% depending on the testing methodology.^15^ This contrasts the proportion of HPV-associated OPSCC of over >60%-70% among North American and Western European populations.^4,14,33-35^ Interestingly, among countries like the United States, the prevalence of HPV-associated OPSCC increased significantly over time; in one study using both p16 IHC and HPV in situ hybridization, the prevalence of HPV association in OPSCC increased from 36% to 72% in men and 29% to 77% in women over 20 years.^33^ An increase was also seen in Denmark with the prevalence rising from 68% to 82% of HPV association in OPSCC over a decade.^36^ More data are needed to assess whether trends will be replicated in sub-Saharan Africa.^37^ Differences in risk factors such as an increased prevalence of HIV, which predispose individuals to a 2-3-fold increased rates of oral HPV infection and 1.5-4 fold increased risk of developing HNSCC,^11,17,18,38-40^ and the global variation in sexual practices such oral sex may influence trends.^41-45^

This study also emphasizes that diagnostics need to be validated in new populations. The testing discordance between p16 and HPV PCR in this study underscores that p16 IHC as a surrogate marker is less reliable among populations with the lowest HPV-AF. In a systematic review, cases from Canada with the highest HPV-AF of 72% had 1.5% discordance compared with countries like Spain with the lowest HPV-AF of 8% and discordance of 30%.^32^ The testing discordance has led to updated recommendations in the College of American Pathologists guideline to performed HPV-specific testing (eg, DNA PCR) in geographic regions with a low prevalence of HR-HPV–associated OPSCC and that p16 is not a suitable standalone surrogate in geographic regions where the HPV AF is <50% and certainly where it is 20% or lower.^31^ Given the lower prevalence of HPV association such as in this cohort, other mechanistic pathways may lead to upregulation of p16 and underly false-positive samples.^46^ Additionally, limitations exist with the specimens and testing itself, including the possibility of small biopsies, biopsies with variations in tumor tissue, and IHC staining technical issues. Finally, tumor biology may also differ between geographic populations. Overall, this testing discordance emphasizes the need to validate predictive assays in new populations and low and middle-income country (LMIC) health systems. Specifically, for HPV association, perhaps HPV-specific testing, such as HPV PCR, should be performed instead of p16 in countries like Tanzania based on regional epidemiology.

This study has implications in epidemiologic monitoring, vaccine policies, and clinical practice. Given the relatively low current prevalence of HPV-associated HNSCC and OPSCC in this cohort and in regional studies,^15^ there remains a need to monitor prevalence trends, in light of the epidemiologic shifts in other regions.^33,36^ Regarding vaccine policies, there is still a substantial proportion of cases preventable with HPV vaccines. Current studies informing vaccine policies in LMICs mainly focus on cervical cancer and do not incorporate HNC into vaccine model outcomes.^47,48^ Given that access to cancer treatment in LMICs is constrained,^49^ preventative vaccination is pivotal at a public health level.^50,51^ Finally, clinical outcomes data are needed. Currently, in the African Head and Neck Society guidelines, HPV testing is not included under the assumption that testing will not change management.^52^ Furthermore, many clinicians currently do not have access to HPV testing for HNSCC in African countries.^19^ Although may not immediately change clinical care, understanding survival outcomes is needed to assess whether the survival advantage seen in other populations applies to individuals in Tanzania and regionally. This information supports clinician counseling and creates data needed to assess whether de-escalation is applicable, particularly in resource-constrained systems.

This study has multiple limitations. First, this is a limited single-institutional study; replicating this study across multiple countries in the continent could help better define the epidemiologic landscape of HPV-associated HNC in the region. Regarding testing, all sample testing was performed in Tanzania by the research team. Additional testing by an accredited laboratory using IHC or in situ hybridization may have been helpful. Furthermore, sample quality and quantity were barriers to obtaining PCR and IHC results for all samples. In future studies, additional samples may help address this challenge.

This prospectively collected study assessing risk factors for and the prevalence of HPV association in HNCs adds to the limited literature from sub-Saharan African countries. Given the implications in patient prognosis, clinical treatment guidelines, and vaccine policies, additional epidemiologic, clinical outcomes, and vaccine modeling studies are needed.

In conclusion, the prevalence of HPV-associated HNC, specifically of the oropharyngeal subsite, is lower in this Tanzanian cohort compared with western populations. Additional data on epidemiologic trends in sub-Saharan African countries, clinical survival metrics, and vaccine modeling incorporating HNC in outcomes are critically needed given implications in patient-level prognosis, resource allocation, and vaccine policies.

## Data Availability

A data sharing statement provided by the authors is available with this article at DOI https://doi.org/10.1200/GO-25-00596.

## APPENDIX

**TABLE A1:** Agreement Between HPV Status On p16 IHC Test and PCR

No. (overall %)	PCR		Total
	Negative	Positive	
All pairs			
p16 IHC			
Negative	59 (65)	8 (9)	67
Positive	20 (22)	4 (4)	24
Total	79	12	91
Oropharyngeal subsite pairs			
p16 IHC			
Negative	7 (54)	2 (15)	9
Positive	3 (23)	1 (8)	4
Total	10	3	13

Abbreviations: HPV, human papillomavirus; IHC, immunohistochemistry; OSCC, oropharynx squamous cell carcinoma; PCR, polymerase chain reaction.

**TABLE A2:** Agreement and Utility of p16 IHC Compared With HPV PCR Testing

Patient Population	Pairs	Concordance (95% CI)	Kappa (95% CI)^a^	McNemar, *P* Value	Sensitivity (95% CI)^a^	Specificity (95% CI)^a^	PPV (95% CI)^a^	NPV (95% CI)^a^
All HNSCC	91	69.2% (59.8 to 78.7)	5.6 (–14.3 to 25.6)	.023	33.3 (6.7 to 60.0]	74.7 (65.1 to 84.3)	16.7 (1.8 to 31.6)	88.1 (80.3 to 95.8)
OPSCC only	13	61.5% (35.1 to 88.0)	3.0 (–51.1 to 57.1)	.655	33.3 (0 to 86.7)	70.0 (41.6 to 98.4)	25.0 (0 to 67.4)	77.8 (50.6 to 100)

Abbreviations: HPV, human papillomavirus; IHC, immunohistochemistry; NPV, negative predictive value; PCR, polymerase chain reaction; PPV, positive predictive value.

^a^ Asymptotic normal standard error confidence intervals.

**TABLE A3:** Occupational and Household Exposures by HPV Status According to PCR Among 121 Patients With SCC

Exposure	Total, No. (%)	Positive, No. (%)	Negative, No. (%)	Unknown, No. (%)	*P* ^a^
	121 (100)	14 (12)	82 (68)	25 (21)	
Chemical	65 (54)	8 (57)	42 (51)	15	.682
Smoke	66 (55)	7 (50)	46 (56)	13	.672
Wood dust	27 (22)	4 (29)	16 (20)	7	.440
Paint fume	25 (21)	3 (21)	16 (20)	6	.868
Asbestos	7 (6)	0 (0)	4 (5)	3	.399
Cook Location					.334
Inside only	70 (58)	10 (71)	48 (59)	12	
Outside only	47 (39)	3 (21)	32 (39)	12	
In and outside	4 (3)	1 (7)	2 (2)	1	
Materials for cooking inside					
Smoke	61 (86)	8 (80)	42 (88)	11	.532
Firewood	69 (57)	5 (36)	45 (55)	19	.185
Charcoal	76 (63)	10 (71)	51 (62)	15	.507
Gas	38 (31)	6 (43)	28 (34)	4	.529
Electric	1 (1)	0 (0)	1 (1)	0	.678
Other	3 (2)	0 (0)	2 (2)	1	.555

Abbreviations: HPV, human papillomavirus; PCR, polymerase chain reaction; SCC, squamous cell carcinoma.

^a^ Pearson chi-squared tests are calculated using only known values.

**TABLE A4:** Oral Health Exposures By HPV Status According to PCR Among 121 Patients With SCC

Dental Factors	Total, No. (%)	Positive, No. (%)	Negative, No. (%)	Unknown, No. (%)	*P* ^a^
	121 (100)	14 (12)	82 (68)	25 (21)	
Dentures	6 (5)	0 (0)	2 (2)	4	.555
Fluorosis	36 (30)	3 (21)	24 (29)	9	.547
Number of missing teeth; median (IQR)	2 (0-4)	1 (0-2)	2 (0-4)	3 (2-4)	.162
Brush teeth	120 (100)	14 (100)	81 (100)	25	NC
Brushing times a day; median (IQR)	1 (1-2)	2 (1-2)	1 (1-2)	1 (1-2)	.384
Tooth cleaning by brush	101 (83)	14 (100)	67 (82)	20	.081
Brush morning	114 (97)	13 (100)	77 (96)	24	.478
Brush afternoon	2 (2)	0 (0)	2 (2)	0	.564
Brush evening	44 (38)	7 (54)	26 (32)	11	.136
Brush after meals	2 (2)	0 (0)	1 (1)	1	.685
Dental pain					.843
Very often	1 (<1)	0 (0)	1 (1)	0	
Fairly often	19 (16)	1 (8)	15 (19)	3	
Occasionally	46 (39)	6 (46)	28 (35)	12	
Hardly ever	23 (19)	3 (23)	16 (20)	4	
Never	29 (25)	3 (23)	20 (25)	6	
Gum bleed					.128
Very often	0 (0)	0 (0)	0 (0)	0	
Fairly often	18 (16)	0 (0)	15 (19)	3	
Occasionally	32 (28)	5 (42)	18 (23)	9	
Hardly ever	23 (20)	4 (33)	14 (18)	5	
Never	43 (37)	3 (25)	32 (41)	8	
Dental function					.845
Very often	2 (2)	0 (0)	1 (1)	1	
Fairly often	5 (4)	1 (7)	4 (5)	0	
Occasionally	34 (28)	2 (14)	22 (27)	10	
Hardly ever	49 (40)	6 (43)	33 (40)	10	
Never	31 (26)	5 (36)	22 (27)	4	
Dental health					.234
Poor	18 (15)	0 (0)	12 (15)	6	
Fair	48 (40)	6 (43)	31 (38)	11	
Good	44 (37)	8 (57)	32 (39)	4	
Very good	8 (7)	0 (0)	7 (9)	1	
Excellent	1 (<1)	0 (0)	0 (0)	1	
Unknown	2	0	0	2	

Abbreviations: HPV, human papillomavirus; PCR, polymerase chain reaction; SCC, squamous cell carcinoma.

^a^ Pearson chi-squared or Kruskal-Wallis tests are calculated using only known values.

**Table A5:** Associations Between HIV Status and HPV Status Differ by Sex

HPV Status	HIV positive	HIV negative	OR (95% CI)	*P*
		Men		
HPV positive	2 (33)	5 (8)	5.7 (0.8 to 39.2)	.053
HPV negative	4 (67)	57 (92)	1	Reference
		Women		
HPV positive	0 (0)	7 (33)	NC	NC
HPV negative	5 (100)	14 (67)		
		Overall		
HPV positive	2 (18)	12 (14)	1	Reference
HPV negative	9 (82)	71 (86)	1.3 (0.3 to 6.8)	.746

Abbreviations: HPV, human papillomavirus; NC, not calculable; OR, odds ratio.
